# Pulmonary effects of e-liquid flavors: a systematic review

**DOI:** 10.1080/10937404.2022.2124563

**Published:** 2022-09-25

**Authors:** Felix Effah, Benjamin Taiwo, Deborah Baines, Alexis Bailey, Tim Marczylo

**Affiliations:** a Pharmacology Section, St George’s University of London, London, UK; b Radiation, Chemical and Environmental Hazards, UK Health Security Agency, Didcot, UK; c Physiology Section, St George’s University of London, London, UK; d Infection and Immunity Institute, St George’s University of London, London, UK

**Keywords:** Electronic cigarette, e-liquid, flavors, lung, toxicity

## Abstract

Electronic cigarettes (ECs) are purported to be tobacco harm-reduction products whose degree of harm has been highly debated. EC use is considered less hazardous than smoking but is not expected to be harmless. Following the banning of e-liquid flavors in countries such as the US, Finland, Ukraine, and Hungary, there are growing concerns regarding the safety profile of e-liquid flavors used in ECs. While these are employed extensively in the food industry and are generally regarded as safe (GRAS) when ingested, GRAS status after inhalation is unclear. The aim of this review was to assess evidence from 38 reports on the adverse effects of flavored e-liquids on the respiratory system in both *in vitro* and *in vivo* studies published between 2006 and 2021. Data collected demonstrated greater detrimental effects *in vitro* with cinnamon (9 articles), strawberry (5 articles), and menthol (10 articles), flavors than other flavors. The most reported effects among these investigations were perturbations of pro-inflammatory biomarkers and enhanced cytotoxicity. There is sufficient evidence to support the toxicological impacts of diacetyl- and cinnamaldehyde-containing e-liquids following human inhalation; however, safety profiles on other flavors are elusive. The latter may result from inconsistencies between experimental approaches and uncertainties due to the contributions from other e-liquid constituents. Further, the relevance of the concentration ranges to human exposure levels is uncertain. Evidence indicates that an adequately controlled and consistent, systematic toxicological investigation of a broad spectrum of e-liquid flavors may be required at biologically relevant concentrations to better inform public health authorities on the risk assessment following exposure to EC flavor ingredients.

## Introduction

Tobacco smoking remains the leading cause of preventable deaths globally (World Health Organization 2019). Tobacco smoke contains approximately 7000 different chemicals, including nicotine (National Center for Chronic Disease Prevention and Health Promotion (US) Office on Smoking and Health 2014), of which 93 chemicals of concern are proposed to produce direct/ indirect harm through inhalation, ingestion, or absorption into the body (FDA 2012; Hoffmann and Hoffmann 1997). Some of these toxicants are responsible for the onset of life-threatening medical conditions that affect the cardiovascular (Frost-Pineda et al. 2011), respiratory (Buist, Vollmer, and McBurnie 2008; Vestbo et al. 2013), and digestive (Doll et al. 2005; El-Zayadi 2006) systems. Nicotine is responsible for tobacco dependence; thus, nicotine replacement therapies (NRT), void of most of the 93 chemicals of concern, are used as smoking cessation aids. It is noteworthy that addition smoking cessation aids also contain low levels of volatile organic compounds (VOCs) which may initiate adverse health effects (Kim, Kim, and Shin 2022). Further, traditional NRTs have protracted nicotine absorption profiles that take several min to reach peak plasma concentrations, creating an unpopular choice for cigarette smoking cessation aids. Alternatively, electronic cigarettes (ECs) or electronic nicotine delivery systems (ENDS) are battery-powered devices designed to vaporize a nicotine-containing solution (known as e-liquid) for a relatively rapid and efficient nicotine delivery system to the brain, which is more comparable to traditional cigarettes. E-liquids contain propylene glycol (PG) and/or vegetable glycerin (VG) and may contain nicotine and/or flavors. ECs, provide reinforcing sensory and behavioral cues in addition to nicotine, which (1) aids in alleviating withdrawal symptoms, (2) reduce nicotine craving, and (3) decreases relapse to smoking (Wadgave and Nagesh 2016).

Nicotine in e-liquids may exist in two distinctive forms: free-base nicotine or nicotine salts, and the state of one or the other is determined by the pH of the e-liquid (Duell, Pankow, and Peyton 2020). Interestingly, the pharmacokinetics of ECs with nicotine salts typically match the absorption profile of a traditional pyrogenic cigarette, thus making them the most utilized non-tobacco nicotine delivery systems in the UK (ASH 2021).

**Figure 1.**
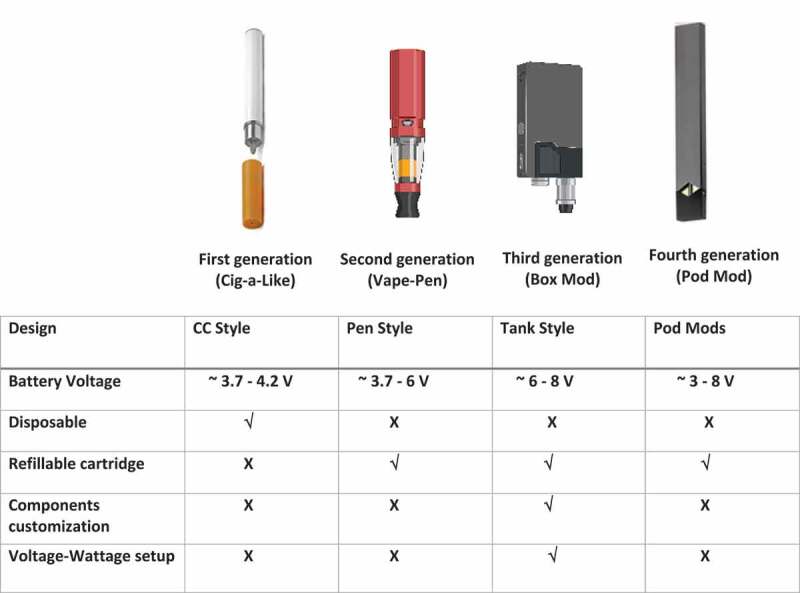
Different generations of EC devices and their main differences. The figure was produced with Biorender.com.

There are many variations of EC devices on the market which are characterized into 4 generations based upon the device’s structure, reusability, and voltage (Figure 1). First-generation ECs are disposable devices that resemble cigarettes. Certain types resemble pens, screwdrivers, or hookah tips, aiming to imitate the conventional way of smoking (Hiemstra and Bals 2016). Compared to the first generation, the second generation is slightly larger in shape with a higher voltage lithium battery, and comprised of a heating element called an atomizer used to heat e-liquids into vapor (i.e. aerosols) and a replaceable cartridge or tank to hold refill solutions (Talih et al. 2015). Third-generation EC devices are comparable to second-generation; however, these provide the user more freedom to customize components of the device (i.e., low resistance ‘sub-ohm’ heating coils) and adjust temperature and power. Low resistance, high power settings enable vapers to ‘cloud-chase’ – consuming greater volumes of e-liquid to generate higher amounts of aerosol. Finally, the fourth generation of ECs is referred to as pod mods, which resemble some of the first EC devices in shape; however, these items are refillable and rechargeable using a USB cable. These e-liquids predominantly contain nicotine salts permitting a quicker nicotine delivery to the brain.

### Electronic cigarette use, recent trends, effectiveness & potential hazards

In 2021, after 16 years of commercial availability, an estimated 3.6 million adults comprising ex-smokers (64.6%) or dual users (30.5%) and relatively few never-smokers (4.9%) were EC consumers in Great Britain (ASH 2021). An increase in the proportion of ex-smokers and a fall in current smokers using EC over time is thought to be due to the general perception that ECs are a safer alternative to tobacco smoking and therefore used as a smoking cessation aid (ASH 2020). A review commissioned by Public Health England of available evidence suggested that ECs are at least 95% less harmful to health than tobacco smoking (McNeill et al. 2018). Therefore, smokers who switched completely to EC-only use are expected to improve their long-term health compared with those that continue to smoke (McNeill et al. 2018).

Although ECs are considered less hazardous than cigarettes, it is less clear how harmful EC use as evidenced by oncogenicity (Bracken-Clarke et al. 2021) is compared with not using EC, especially in former smokers. EC users avoid or reduce exposure to most of the 93 toxicants in combusted tobacco smoke. It is considered that approximately 80% of cigarette smoke-induced cardiovascular toxicity is attributed to the first 1–3 cigarettes per day (Gallucci et al. 2020). This is likely in contrast to vaping because carbon monoxide (CO) and many other toxicants are not generated during vaping. However, in addition to nicotine, EC users are also exposed to PG, VG, flavor compounds, VOCs and both degradation and condensation byproducts formed during the heating of e-liquid to generate an inhalable aerosol (Kim, Kim, and Shin 2022; Schick et al. 2017). It appears that the composition of the e-liquid and repeated dosing or vaping “behavior” may be responsible for the observed toxicity. Burgwardt et al. (2020) reported that Valsalva vaping may enhance exposure to harmful constituents such as flavoring in the EC aerosol vaper. Valsalva vaping is achieved when EC users over-inhale and then forcefully exhale to eliminate the vapor, which is usually produced in greater quantities than that generated by a conventional cigarette. Concerns exist that EC use may be associated with the onset of various adverse health effects related to the cardiovascular (Bhatnagar 2016; Schweitzer, Wills, and Behner 2017) and lung diseases (Chun et al. 2017; Moazed and Calfee 2017). Data in animals suggest that short-term or long-term exposures to EC aerosols might result in cardiovascular remodeling driven by PG/VG vehicles, with nicotine potentially playing a protective role (Neczypor et al. 2022). Analyses of the variety and concentrations of EC aerosol toxicants infer that EC aerosol is less harmful than tobacco smoke (Azzopardi et al. 2016); however, the presence of lower concentrations of tobacco-related toxicants indicates these substances are not likely to be risk-free.

Several investigators reported the presence of hazardous carbonyl compounds such as formaldehyde, acetaldehyde, glyoxal, and acrolein in EC aerosol (Bekki et al. 2014; Etter, Zäther, and Svensson 2013; Ogunwale et al. 2017; Schober et al. 2014) and tobacco-specific N-nitrosamines (TSNA) in e-liquids and their aerosols (Farsalinos et al. 2015). Acetaldehyde and formaldehyde are recognized carcinogens (Feron et al. 1991; Roe and Wood 1992), and acrolein is a cardiovascular toxicant (Arman and İşisağ Üçüncü 2020; DeJarnett et al. 2014). Tobacco-specific N-nitrosamines are carcinogenic compounds formed from the curing and processing of tobacco leaves through nitrosation of nicotine and related tobacco alkaloids (Bush et al. 2001; Wang et al. 2017). Conflicting evidence exists from (Farsalinos et al. 2015; Goniewicz et al. 2014a; Kim and Shin 2013; Laugesen 2008; U.S. Food and Drug Administration 2009) to suggest that ECs are a source of TSNA exposures; however, it is physiologically possible that endogenous formation might originate from nicotine through nitrite exposure in the saliva (Knezevich et al. 2013), acidic stomach, or microbiome-mediated nitrosation at different sites (Ziebarth 1997). Traces of toxic metals such as cadmium, nickel, and lead originating from EC devices (Goniewicz et al. 2014a) are also found in the blood and urine of EC users (Aherrera et al. 2017; Badea et al. 2018; Goniewicz et al. 2018; Jain 2019;). Exposure to these metals is known to induce nasal and lung cancers and developmental defects (Hsueh et al. 2017; Jaishankar et al. 2014; Son 2020). Further, EC refills and aerosols contain volatile organic chemical compounds like benzene, toluene, xylene, and styrene (BTXS) (Schober et al. 2014), albeit at substantially lower concentrations than in tobacco smoke (Margham et al. 2016). Previous epidemiological studies found that individuals who are chronically exposed occupationally to BTXS (individuals or mixtures) exhibit a lower peak expiratory flow [PEF, litter per second (L/s)] and 25–75% of forced expiratory flow (FEF25∼75%, L/s) (Liao et al. 2022). In addition, occupational exposure to BTXS was linked to risks of prostate (Blanc-Lapierre, Sauvé, and Parent 2018) and lung cancer (Warden et al. 2018). In addition to this evidence, concerns over increasing utilization by young individuals and the potential for youth EC use to progress to tobacco smoking led to regulations restricting their use in many countries. In the UK alone, there has been a 3% rise in EC use among young people between the ages of 11–17 from 2020 to 2022 (ASH 2022). Although there are some regulations regarding the use of EC in the EU, the Tobacco Products Directive 2014/14/EU (TPD) introduced new restrictions on nicotine concentration and size of EC refill containers, the government needs to ensure existing laws are enforced and identify where regulations could be extended.

### Electronic cigarette flavorings

The flavoring compounds used in EC e-liquids are generally regarded as safe (GRAS) for ingestion and employed extensively in the food industry. However, there are concerns regarding EC flavoring toxicity following inhalation using EC as evidence for safety following has not been extensively investigated (Farsalinos et al. 2013; Khlystov and Samburova 2016; Leigh et al. 2016; Sundar et al. 2016). In 2014, there were an estimated 8000 flavored e-liquids on the market, which has likely increased significantly (Zhu et al. 2014). Initially, the most popular EC flavors in the UK mimicked the flavors of tobacco products including cigarettes, cigars, or pipe tobacco and menthol (ASH 2021). However, preferences have shifted more recently to fruity flavors, and the popularity of other flavors such as desserts, candy or beverages has also risen (ASH 2021). Unfortunately, some product names and flavors provide little information regarding the flavor category such as unicorn blood, truth serum or snake oil let alone their likely constituents. The vast number of products and presence of hard-to-categorize product names led the Dutch National Institute for Public Health and the Environment (RIVM) to develop a flavor wheel (Krüsemann et al. 2019),which groups e-liquids into 13 flavor categories: candy, dessert, other sweets, fruit, alcohol, coffee/tea, other beverages, spices, nuts, menthol/mint, tobacco, and unflavored, based upon the chemical composition of the e-liquid. Table 1 presents the main flavoring chemicals in several flavors in e-liquids.

**Table 1.** Flavors in e-liquid and some of the main flavoring chemicals they are composed of.

Flavoring chemical	Common flavors with chemical agents	References
Cinnamaldehyde, 2-methoxycinnamaldehyde	Cinnamon	(Behar et al., 2014; Gerloff *et al*., 2017)
3,4-Dimethoxybenzaldehyde	Cherry	(Kavvalakis *et al*., 2015)
2,5-dimethylpyrazine	Chocolate	(Kavvalakis *et al*., 2015)
Maltol, ethyl maltol	Caramel, Vanilla	(Gerloff *et al*., 2017; Tierney *et al.,*^2016^)
Menthol, Menthone	Mint	(Berkelhamer *et al*., 2019)
p-Anisaldehyde	Anise	(Behar *et al*., 2018a; Berkelhamer *et al*., 2019; Noël et al. 2020b)
Benzaldehyde	Almond	(Behar *et al*., 2018a; Berkelhamer *et al*., 2019)
Vanillin, O-vanillin	Vanilla	(Behar *et al*., 2014; Gerloff *et al*., 2017; Muthumalage et al. 2018)
Hexyl acetate, 3-Hexen-1-ol	Apple	(Kerasioti *et al*., 2020; Kim *et al*., 2018)
Isoamyl acetate	Banana	(Fetterman *et al*., 2018)
Methyl cinnamate	Balsamic, Strawberry	(Berkelhamer *et al*., 2019; Leigh *et al.,*^2016^)
Methyl anthranilate	Berry	(Tierney et al. 2016)
Other flavoring agents less known: Damascenone (α or β), 3-methyl-1,2- cyclopentanedione, acetamide, linalool, terpineol, citral, corylon, anisaldehyde, trimethylpyrazine, eugenol, benzaldehyde, limonene	Miscellaneous	(Gerloff *et al*., 2017; Tierney *et al.*, 2016)

Flavor additives in food may undergo enzymatic metabolism, which might produce fewer toxic metabolites due to phase I and II metabolism (Del Olmo, Calzada, and Nuñez 2017). In contrast, exposure to GRAS-like substances by inhalation after thermal degradation may produce pharmacologically active compounds that produce severe adverse health effects (Fedan et al. 2006). Rose (2017) reported that occupational diacetyl inhalation exposure might lead to bronchiolitis obliterans (BO), more commonly referred to as “popcorn lung.” Bronchiolitis obliterans induced severe coughing, wheezing, and shortness of breath, symptoms resembling chronic obstructive pulmonary disease (COPD) (Aguilar, Michelson, and Isakow 2016). van Rooy et al. (2007) conducted an epidemiological study in the Netherlands suggesting a causal link between BO and chemical workers producing diacetyl for food flavorings. The British Medical Association (BMA) believes flavors’ safety should be closely monitored as evidence for potential adverse effects are emerging after heating and inhalation of e-liquid aerosol (Sassano et al. 2018). The medicines and healthcare products regulatory agency (MHRA) collects UK reports of EC’s harmful effects and safety concerns through their yellow-card scheme (https://yellowcard.mhra.gov.uk/).

The EU TPD established a priority list of 15 potentially harmful additives (Havermans et al. 2022) that needs to not be contained in EC based upon the published literature. These include flavoring additives and chemicals such as guaiacol, geraniol, menthol, ethyl maltol, and diacetyl. Since 2016, diacetyl has also been banned from e-liquids in the UK, whereas flavors containing menthol and ethyl maltol are still available.

Flavors in e-liquids, as mentioned earlier, may contain pharmacologically active compounds (Clapp et al. 2019). Several investigators found that mint flavors contain menthol or menthone (Pereira et al. 2013), and candy flavors including vanillin and cinnamaldehyde (Krüsemann et al. 2019). Fruit flavors might contain 3-hexen-1-ol (apple) or methyl anthranilate (berry), and others (Tierney et al. 2016) (Table 1). Menthone is the ketone analog of menthol. Menthol (l-menthol) is a naturally-occurring agonist for the cold transient receptor potential melastatin 8 (TRPM8), which is functionally expressed in the airways (Sabnis et al. 2008). Due to the efficacy of menthol in inhibiting cigarette-induced cough via the TRPM8, it was added to tobacco as a ‘flavoring’ (Lin et al. 2018). Moreover, the masking of the harshness and irritancy of tobacco underpins the initiation of more intense and protracted smoking (Wickham 2020). This effect contributes to the inclusion of menthol in the EU TPD list of potentially harmful tobacco additives, yet there is a widespread use of menthol in e-liquids.

Vanillin and cinnamaldehyde are TRPV1 and TRPA1 agonists, respectively, which are also functional in the airways (Wang et al. 2019). Therefore, activation of TRPM8, TRPA1, and TRPV1 through EC usage may alter normal physiological responses of the airways. The effects of other chemical constituents of flavors, such as 3-hexen-1-ol and methyl anthranilate, warrant further investigation. Unsurprisingly, the largest category on the flavor wheel, given its popularity in the UK, is fruit, which the National Institute for Public Health and Environment (RIVM) in the Netherlands subcategorized into groups (tropical, citrus, berries, and others) in the flavor wheel (Krüsemann et al. 2019). Not explicitly covered by the flavor wheel, tobacco e-liquids may be sub-divided into those that extract flavor compounds directly from tobacco and those using synthetic flavorings to simulate tobacco taste.

There is little to no direct evidence of the toxicological effects of EC flavors in humans. To date, studies of EC flavor-induced are primarily *in vitro* studies in human cell lines, with a handful of investigations conducted *in vivo*. Therefore, this review aimed to systematically synthesize and critically evaluate published data on flavored e-liquids and their effects on the respiratory system to identify EC flavors and flavor chemicals most associated with adverse respiratory effects. A secondary aim was to investigate whether the outcomes observed *in vitro* are reflected by adverse effects reported *in vivo*. Finally, a potential ‘gold standard’ approach is proposed for future EC investigations regarding the experimental method, which may help reduce inter studies discrepancies. This is the first systematic review to focus specifically on the toxicity of EC flavors on the respiratory system. It may help researchers identify the essential EC flavors and chemicals that need to be the focus of further investigations, including risk assessments that may advise regulators on meaningful and appropriate e-liquid ingredient regulation.

We acknowledge that Stefaniak et al. (2021) published a systematic review with a search engine dating only 8 months prior to our search commencement. Although their review contained a comprehensive description of EC and of published evidence on flavored-e-liquid toxicity across different *in vitro* biological models and *in vivo* systems, our review focuses on the effects specifically on the pulmonary system. Although the Stefaniak et al. (2021) review summarized the published data, our aim was to be more critical and questioning of the published data, including 1) contributions of nicotine and inconsistencies across studies, 2) effect of vehicle/PG/VG, and lack of appropriate controls in many studies 3) potential additive effects of nicotine 4) although a mention of primary cells being more sensitive than immortalized cell lines was made, no differentiation between normal and cancer-derived cell lines was done 5) there was no discussion on the appropriate dose and minimal discussion on the dose-response relationship and improvement this gives to overcoming uncertainty 6) there were little interstudy comparisons 7) there was little explanation for differences in observations between air-liquid interface (ALI) and submerged cultures. It is appreciated that several of these points were raised in their knowledge gaps section, but a critique of the current evidence regarding these gaps was not provided. Therefore, a critical assessment of the literature was undertaken and where the results allow for a misleading interpretation of flavor toxicity, this has been discussed this in detail, which separates our approach significantly from that of Stefaniak et al. (2021).

## Methods

### Data sources and searches

A literature search was completed in November 2020, which was updated in November 2021, using Scopus and healthcare database advanced search (HDAS) (including CINAHL, EMBASE, MEDLINE, PsychINFO, PubMed. Retrieved articles on *in vitro* and *in vivo* studies were published between 2006 and 2021 Keywords included terms to capture concepts associated with e-cigarettes, flavors, and the respiratory system. The search stream here pasted was used for HDAS search – (((((electronic cigarette OR electronic nicotine delivery system OR electronic non-nicotine delivery system OR e cigarette OR e-cigarette OR e-cig OR e cig) AND (flavor OR flavor OR flavoring OR flavoring OR flavoring agents OR Flavoring agents OR Flavored OR Flavored)) AND (lung OR alveolar OR bronchial OR pulmonary)).title, abstract, key. Whereas the search stream below was used for our Scopus search – ((TITLE-ABS-KEY (“electronic cigarette”)) OR (TITLE-ABS-KEY (“electronic nicotine delivery system”)) OR (TITLE-ABS-KEY (“electronic (non) nicotine delivery system”)) OR (TITLE-ABS-KEY (“e cigarette”)) OR (TITLE-ABS-KEY (“e-cigarette”)) OR (TITLE-ABS-KEY (“e-cig”)) OR (TITLE-ABS-KEY (“e cig”))) AND ((TITLE-ABS-KEY (flavor)) OR (TITLE-ABS-KEY (flavor)) OR (TITLE-ABS-KEY (flavoring)) OR (TITLE-ABS-KEY (flavoring)) OR (TITLE-ABS-KEY (“Flavouring agents”)) OR (TITLE-ABS-KEY (“Flavoring agents”)) OR (TITLE-ABS-KEY (“Flavoured”)) OR (TITLE-ABS-KEY (“Flavored”)) AND (TITLE-ABS-KEY (lung)) OR (TITLE-ABS-KEY (alveolar))) AND ((TITLE-ABS-KEY (lung)) OR (TITLE-ABS-KEY (alveolar)) OR (TITLE-ABS-KEY (bronchial)) OR (TITLE-ABS-KEY (pulmonary))) AND (LIMIT-TO (DOCTYPE, “ar”)) AND (LIMIT-TO (LANGUAGE, “English”)))))).

### Selection and exclusion criteria

**Figure 2.**
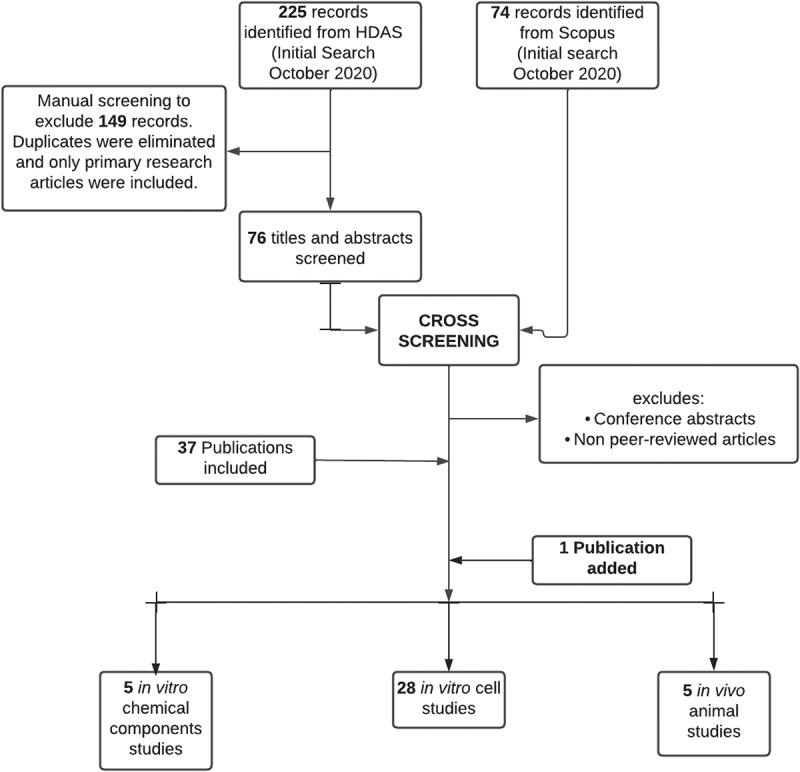
PRISMA flowchart. Articles were retrieved from HDAS and Scopus databases. Primary research articles that met the selection criteria were included irrespective of the year of publication. The figure was created with Lucidchart.com.

Retrieved articles were screened, duplicates were eliminated, and remaining citations were organized in Zotero (George Mason University, Virginia) following Preferred Reporting Items for Systematic Reviews (PRISMA) guidelines. Only primary articles published in English were included. After a manual screening of titles and abstracts within the 225 and 74 records from HDAS and Scopus, respectively, 38 primary articles (one paper outside the search string) met the inclusion criteria for this systematic review (Figure 2).

## Results

The search stream included 28 *in vitro* studies where human cell lines were exposed to flavored e-liquids, 5 articles were e-liquid chemical composition studies, and 5 reported studies where rodents (mice and rats) were exposed to flavored e-liquids. Analysis of the 38 included reports and their reported adverse effects of flavors are summarized in Tables 2, 3 , 4 , 5 , and 6.

**Table 2.** In vitro studies where toxicological effects of flavored e-liquids were investigated with primary epithelial cells alone or together with immortalized normal pulmonary cells.

Cell type	Flavors or flavoring chemicals investigated	Exposure (Time & flavor dose)	E-liquid Nicotine Dose	Nicotine Control	Main findings	Reference
Primary human bronchial epithelial (HBECs), immortalized bronchial epithelial cells (BEAS-2B)	Hot Cinnamon Candies, ‘Sinicide’, Kola (Each containing cinnamaldehyde) Cinnamaldehyde alone at 5 different concentrations (0.05, 0.25, 0.5 & 5 mM).	0, 20, 40, 60, 80, 100, 120 min exposure to aerosol at ALI	No nicotine	N/A	Since aerosol transiently suppressed ciliary frequency beat (CBF), and cinnamaldehyde recapitulated and other differential toxic effects in both cell lines following metabolic and glycolysis assays using a Seahorse XFe21 extracellular flux analyzer. Cinnamaldehyde impaired mitochondrial respiration and glycolysis dose-dependently in BEAS-2B. Reduction of Intracellular ATP levels dose-dependently in differentiated hBE and BEAS-2B cells.	(Clapp et al. 2019)
Human bronchial epithelial cells (hBE)	Apple (3 different variants)	Puff duration of 2.6 s, 50 × 3 s puffs with 5 s in between were bubbled through 10 mL of culture medium to create EC extract. Cells were exposed to 100% of this media.	0, 18 mg/ mL	Nicotine alone controls present	Each apple flavor increased necrosis and apoptosis because of flavor effects. Each apple flavor reduced the secretion of TNF-α, IL-6, IP-10, MIP-1α, and MIP-1β because of flavor-effects. Nicotine alone induced a decrease in pro-inflammatory cytokines (IP-10 & MIP-1β) similar to cigarette smoke extract (CSE) and increased % of macrophages efferocytosing hBE cells but had no effect when in combination with e-liquid.	(Ween et al. 2020a)
Primary human 24-h alveolar macrophages, neutrophils, and natural killer (NK) cells.	Kola Hot Cinnamon Candies Menthol Banana pudding Menthol tobacco Banana ‘Sinicide’	Adherent alveolar macrophages were challenged in triplicate with cell culture medium containing 1, 0.5, or 0.25% flavored e-liquid for 1 h	0, 0.6, 1.2, 1.8, and 2.4 mg/ml	No nicotine alone controls present	Hot Cinnamon Candies, and Kola, and each decreased phagocytosis and viability of macrophages, neutrophils, and NK cells. Hot Cinnamon Candies, and Kola mediated suppression of alveolar macrophage phagocytosis was reversed by coexposure of cinnamaldehyde. No nicotine dose-dependent effects were observed. No nicotine x flavor interaction observed.	(Clapp et al. 2017)
Primary human bronchial epithelial cells	Diacetyl (DA)	1-h ALI aerosol exposure to 12, 25, and 50 mM of diacetyl.	No nicotine	N/A	LDH increased significantly with 50 mM DA- compared to PBS, 12- and 25-mM DA-exposed cultures. Transient decrease in trans-epithelial electrical resistance in all DA-exposed cultures. At 25 and 50 mM, airway basal cell injury with keratin five ubiquitination and decreased p63 expression were observed. The ubiquitin-proteasome-pathway partially mediates airway basal cell repair after acute DA exposure.	(McGraw et al., 2020)
16HBE14o- airway epithelial cell line (16HBE), Normal human bronchial epithelial cells (NHBE), THP-1 differentiated macrophages & primary alveolar macrophages (AM).	Cappuccino Chocolate Peppermint	E-cigarette vapor extracts (EVE) generated using 50 x 3s puffs and exposed to cells for 24-h	18 mg/mL (No nicotine-free e-liquids)	Nicotine alone controls present	Cappuccino, Chocolate and Peppermint caused cellular cytotoxicity by inducing morphological changes and cell shedding in 16HBE. All 10 flavors induced significant LDH release (chocolate highest and nicotine alone significantly higher) in 16HBE. Chocolate > cappuccino >peppermint > banana induced apoptosis. Nicotine and PG:VG did not induce apoptosis in 16HBE and primary HBE, the other cells were not specified). Chocolate flavor most cytotoxic following an LDH assay in NHBE in inducing necrosis and apoptosis. Nicotine showed significant toxicity but lower than chocolate, whereas PG:VG showed no effect. Mango, Tobacco, Banana and Chocolate caused decreased macrophage phagocytic capacity. PG:VG and nicotine alone showed a significant effect on efferocytosis. Chocolate > Banana > nicotine induced increase in IL-8 secretion by NHBE. Decrease in IL-1β by all flavors as well as nicotine. Effects are linked to concentration and benzene-ring containing chemicals.	(Ween et al. 2020b)
Human bronchial epithelial cells of COPD models, immortalized 16HBE cells	Virginia Tobacco (Flavor from tobacco extract) Menthol	Intense puffing regime of 55 mL over 4 s, with 30s rest interval) for 30 min at ALI	5 mg/ml	No nicotine alone control	Virginia tobacco-induced significantly higher LDH levels compared to menthol in the COPD cells Menthol in 16HBE induced a significantly lower LDH release when compared to air-only control. No difference in DNA damage in either cell line in Virginia tobacco or menthol when compared to cigarette controls. In COPD cells, cigarette, and Virginia tobacco-induced a significant decrease in IL-8 production. No alteration in IL-6 release compared to air-only controls. In 16HBE, IL-8 production was significantly higher in all groups compared to air-only controls. Tobacco significantly decreased IL-6 release compared to cigarette.	(O’Farrell et al. 2021)

**Table 3.** In vitro studies where toxicological effects of flavored e-liquids were carried out on immortalized pulmonary normal epithelial cells alone or together with carcinoma pulmonary cells.

Cell type	Flavors or flavoring chemicals investigated	Exposure (Time & flavor dose)	E-liquid Nicotine Dose	Nicotine Control	Main findings	Reference
Human bronchial epithelial cell line (16HBE14o)	2,5-dimethylpyrazine (chocolate, nutty flavor) Damascenone (apple, citrus, wine-like) Linalool (floral, spice) α-ionone (fruity, raspberry) Ethyl maltol (caramel) Furaneol (strawberry, sweet) Vanillin (vanilla)	24-h direct exposure (flavoring chemical)	No nicotine	N/A	2,5-Dimethylpyrazine reduces the physiological response to signaling molecules important in aspects of airway epithelial cell innate immunity. 2,5-Dimethylpyrazine increases airway epithelial ion conductance. 2,5-Dimethylpyrazine activates apical ion efflux via cystic fibrosis transmembrane conductance regulator (CFTR). Vanillin and 2,5-Dimethylpyrazine significantly altered airway epithelial cellular physiology indicative of cellular signaling events. Linalool, α-ionone, damascenone, ethyl maltol, and furaneol, no significant effects?	(Sherwood and Boitano, 2016)
Immortalized human bronchial epithelial cells (16-HBE, BEAS-2B)	Cool Cucumber Mango Strawberry coconut Classic Menthol Caffe Latte	66 puffs during 22 minutes with a three-second puff duration at 1.6 L/min flow rate and an inter puff interval of approximately 17s.	50 mg/mL nicotine	No nicotine control	Cool cucumber, classic menthol, caffe latte induced pro-inflammatory biomarkers (IL-1ß, IFN-γ, IL-17, etc.) depending on the cell line as 16-HBE and BEAS-2B present specific physiological differences. It is unclear whether these effects are in part influenced by nicotine content due to the lack of nicotine control samples. Classic Menthol induced the most significant mitochondrial ROS production in 16-HBE. Prostaglandin production was induced in BEAS-2B, not in 16-HBE, by strawberry coconut. IL-8 production was induced in 16-HBE, but not in BEAS-2B, by cool cucumber, classic menthol, strawberry coconut, and caffe latte.	(Muthumalage et al. 2019)
Normal human bronchial epithelial (NHBE) cells	Flavoring chemicals diacetyl and 2,3-pentanedione (Concentrations of diacetyl and 2,3-pentanedione were not specified)	24-h chemical exposure after media dilution in an ALI model	No nicotine	N/A	Cytoskeletal and cilia processes among common genes (142 genes) were perturbed by both diacetyl and 2,3-pentanedione. The expression of multiple genes involved in cilia biogenesis was significantly downregulated by diacetyl and 2,3-pentanedione. The number of ciliated cells significantly decreased.	(Park et al., 2019)
BEAS-2B and human pulmonary fibroblast (HPF)	Ethyl maltol Menthol	48-h chemical (diluted liquid) direct exposure	No nicotine	N/A	Both flavor chemicals were highly cytotoxic following a MTT assay at concentrations 30 (menthol) and 100 times (ethyl maltol) lower than the highest concentrations in the refill fluids. BEAS-2B cells (IC50 = 0.15 mg/ml) were more sensitive to ethyl maltol than hPF (IC50 = 0.28 mg/ml). No difference in sensitivity to menthol between HPF and BEAS-2B.	(Omaiye et al. 2019)
BEAS-2B	Menthol Virginia Tobacco	30 min x 3 (Each one after 12-h) aerosol exposure	5% nicotine (Specific mg/ml of nicotine not specified)	No nicotine control	Menthol- mitochondrial effects: proton leakage, a decrease in coupling efficiency, and a reduction of complex I, II, and IV 24-h post-menthol exposure reduction of basal respiration, maximal respiration, spare capacity, and complex I. Tobacco had no significant effects on mitochondrial respiration, but immediately post final exposure increased complex I, IV, and V. Whether this effect is synergistic with nicotine was unclear.	(Lamb, Muthumalage, and Rahman 2020)
Human pulmonary fibroblasts (HPF)	Cinnamon Ceylon	48-h e-liquid direct exposure to 1% and 0.3% doses.	Different concentrations of nicotine, but not specified.	No nicotine control	Nicotine concentration did not correlate with cytotoxicity in MTT assays. Cinnamon Ceylon cytotoxicity effect following MTT assay was dose-dependent. Cinnamaldehyde and 2-methoxycinnamaldehyde (2-MOCA) were the major components of cinnamon flavors. Cinnamaldehyde and 2-MOCA standards were highly cytotoxic in MTT assay.	(Behar et al. 2014b)
Human lung fibroblasts (HPF)	Bubblegum Butterscotch Caramel Butterfinger Menthol Artic Wisconsin frost Domestic JC Original French vanilla Vanilla Tahiti Tennessee cured Island Valencia Mint chocolate Swiss Dark Caramel Espresso Mercado Simply strawberry Arctic Menthol Summer Peach Black Cherry Chocolate truffle Cinnamon Ceylon	48-h direct e-liquid exposure to 0.001%, 0.01%, 0.03%, 0.1%, 0.3%, and 1% v/v.	0 to 24 mg/ml	No nicotine alone control	Most significant cytotoxicity following MTT assay was observed with cinnamon and menthol flavors which did not contain nicotine. Levels of nicotine were not correlated with the degree of MTT cytotoxicity. The humectants vegetable glycerin (VG) and propylene glycerin (PG) were non-cytotoxic for the hPF.	(Bahl et al. 2012)
Human airway epithelial cells (H292) & human lung fibroblast cells (HFL-1 cell line)	Tobacco Cinnamon roll Grape	30 s interval with a 4 s pulse for different time durations 5, 10, and 15 minutes at ALI	0, 6, 16, 18 and 24 mg	No nicotine alone control present	Tobacco and cinnamon roll-flavored e-liquid increased the secretion of inflammatory cytokines IL-6 and IL-8 in H292 and HFL-1. The addition of nicotine gave a striking, dose-dependent increase only in IL-8 secretion in HFL-1.	(Lerner et al. 2015)
Normal human bronchial epithelial (NHBE) cells	Menthol Tobacco Vanilla Blueberry	24-h direct e-liquid exposure	Different dilutions (0.03125%, 0.0625%, 0.125%, 0.25%, 0.5%, 1%, 2% & 3% of 24 mg/ml e-liquids.	No Nicotine control	Only menthol elicited dose-dependent cytotoxic effects on activating G0/G1cycle arrest, decreasing glutathione concentration, activation of caspase 3/7 activity, increasing nuclear factor-kB (NF-kB), & increased mitochondrial mass. Tobacco, vanilla, and menthol decreased mitochondrial membrane potential dose-dependently. Glutathione content decreased after exposure to tobacco, blueberry, and vanilla, but only above 1%. No nicotine-free e-liquids were tested. Therefore, it is unclear whether these effects are due to flavors alone or a combination of flavor plus nicotine. Base liquids (PG/VG), with or without nicotine, and commercial, flavored, nicotine-containing e-liquids (CFs), had little or no effect on cell viability, cell count, mitochondrial mass, and potential and oxidative stress.	(Czekala et al. 2019)
Pulmonary fibroblasts (HFL-1)	A mixture of tobacco, coconut, vanilla, and cookie	24-h direct e-liquid exposure 0.1%, 0.25%, 0.5% & 1%.	3 mg/ml (No nicotine-free group included)	Nicotine alone control present at 0.25% and 0.5%.	A dose-dependent decrease in cell viability using the mixture of e-liquids. An increase in IL-8 release induced only by 0.25% flavor mix. Elevated senescence-associated beta-galactosidase (SA-β-gal) activity in a dose-response fashion. Transforming growth factor-β1 (TGF-β1) induced myofibroblast differentiation was inhibited by flavor mix, decreasing α-smooth muscle actin and fibronectin protein levels at only one concentration 0.5%. No significant nicotine effect was observed.	(Lucas et al. 2020)
Human bronchial epithelial cell lines (BEAS-2B, H292)	Crème brûlée-favor	5s duration every 30s for 1-h to aerosol at ALI	50 mg/mL	No nicotine control	Aerosol decreased cell viability (≥50%) of BEAS-2B cells alone and increased nitric oxide (NO) production (≥30%), as well as iNOS gene expression. In H292 cells, aerosol increased the production of reactive oxygen species (ROS). Aerosol dysregulated expression of several genes related to biotransformation, inflammation, and airway remodeling, including CYP1A1, IL-6, and MMP12 in both cell lines. Because nicotine-free e-liquids were not investigated, it is unclear whether nicotine has any synergistic effects with Crème brûlée-flavor.	(Pinkston et al. 2020)
BEAS-2B, Human cystic fibrosis cell line (IB3-1), and Calu3	Apple, Cherry, Tobacco, Strawberry	24-h exposure to condensates (50%, 25%, and 12.5% diluted with media)	0, 6, 12 and 18 mg/mL	No nicotine alone control	Cherry- similarly cytotoxic to the C38 and BEAS-2B In BEAS-2B, tobacco- also cytotoxic following cell viability at all dilutions tested. Strawberry reduced cell viability in BEAS-2B cells was significantly greater than apple, cherry, and tobacco CALU-3 showed no cytotoxic response to any flavor. There was no nicotine concentration-dependent effect.	(Leslie et al. 2017)
Human pulmonary fibroblasts (HPF), alveolar epithelial cells (A549)	Vanilla Mint/Menthol Butterscotch Caramel Butterfinger	E-fluid (0.001%, 0.01%, 0.03%, 0.1%, 0.3%, 1.0%) and aerosol exposures. For each batch of aerosol, 24 puffs were collected into 4 mL of culture medium	18 mg/ml	No nicotine control	Vanilla (creamy/buttery) and Butterscotch (creamy/buttery) were cytotoxic following MTT assay both as fluid and as aerosol. Mint/Menthol was cytotoxic following MTT assay only as e-liquid. Butterfinger (creamy/buttery) and caramel aerosol were the most toxic in a dose-dependent fashion of all flavors following MTT assays. It is unclear whether the effects observed are due to nicotine plus as there are no nicotine controls.	(Behar *et al*., 2018)

**Table 4.** *In vitro* studies where toxicological effects of flavored e-liquids were investigated in pulmonary carcinoma cells alone or together with primary pulmonary cells.

Cell type	Flavors or flavoring chemicals investigated	Exposure (Time & flavor dose)	E-liquid Nicotine Dose	Nicotine Control	Main findings	Reference
Human bronchial epithelial cells (H292)	Strawberry Tobacco Pina colada Menthol Coffee	3 s puff duration, every 30s, with a 55 mL puff volume. A period of 30 min was used as this was the minimum exposure at ALI.	0, 6, 12, 18, or 24 mg/mL of nicotine	Nicotine alone control present.	Strawberry flavor was most cytotoxic following neutral red assay. Exposure to each aerosol decreased metabolic activity and cell viability. Coffee increased the release of IL-6, CXCL1, and CXCL2. Strawberry increased the release of IL-1ß IL-10, CYCL1, CXCL2, and CXCL10. No significant differences were observed between the different concentrations of nicotine and air control for both metabolic activity and viability. Unflavored e-liquids significantly decreased in IL-1β, CXCL1, and CXCL2 in response to 18 mg/mL nicotine aerosol versus air controls. Unflavored e-liquids significantly increased IL-6 in response to 24 mg/mL nicotine aerosol versus air control. Metabolic activity was significantly decreased in cells exposed to PG/VG and VG-only, but not PG-only aerosols compared to air controls.	(Leigh et al. 2016)
Primary bronchial epithelial cells and human distal lung epithelial cells (NCI-H441)	Mix-1: raspberry, orange, lemon, and lime Mix-2: ripe strawberry, sweet apples, and tart kiwi	40 ml/puff, 3s puff duration, 30s puff interval, 10 puffs/ session at ALI	0, 3 mg/ml	No nicotine alone control present	Exposure to mix-1 (nicotine-free) significantly increased transcript expression of pro-inflammatory cytokines CXCL8, IL6, NFKB1, TNF (not specified whether α or β ) by more than three-fold and IL-1ß by 44-fold. Mix-2 (nicotine-free) PI3 transcript was reduced two-fold. The presence of nicotine in mix-1 significantly reduced the release of inflammatory markers of IL-1ß, 1 L-10, IL-13, and IL-6. The presence of nicotine in mix-2 increased the transcript of TNF (not specified whether α or β)	(Ganguly et al. 2020)
H292 cells	Cinnamon Butter	2-h/day for 1 or 3 consecutive days aerosol exposure. Using sub-ohm EC	mg/mL	No nicotine control	1 Day of exposure is cytotoxic, alters gene expression, and increases reactive oxygen species (cinnamon> butter). After 3 days of exposure, cinnamon flavor is more cytotoxic following LDH assay and causes oxidative damage to a greater extent than butter-flavored e‑cig aerosol. Cinnamon-flavored aerosol induced pro-oxidant effects, e.g., increased 8-hydroxy-2-deoxyguanosine levels. It is unclear whether the effects observed are due to nicotine plus as there are no nicotine controls.	(Noël et al. 2020b)
A549 (3D) co-culture of alveolar and H441 cells	Menthol Cinnamon Tobacco	24-h exposure to condensed aerosol (puff regime was set to 55 mL puff volume, 3 s draw, 60s 200 puff)	0 mg/ mL & 18 mg/ mL	No Nicotine control present	Cell viability significantly decreased in mono- and 3D culture by mint and cinnamon. Significant IL-8 release only in monoculture after Cinnamon exposure. No increase in MCP-1 release E-liquids containing nicotine did not alter cytotoxicity or pro-inflammatory effects relative to nicotine-free.	(Bengalli et al. 2017)
CALU-3 airway epithelial cells	Banana Pudding (Southern Style) Kola Hot Cinnamon Candies Menthol Tobacco Solid Menthol Captain black cigar Peanut butter cookies Popcorn Black Licorice Vanilla Tobacco	24-h direct e-liquid exposure (0%, 0.1%, 0.3%, 1%, 3.0%, 6.0%, 10% (v/v))	0 mg/mL and 12 mg/mL of nicotine	Nicotine alone control present	Hot cinnamon candies and menthol tobacco show cytotoxicity (cell and mitochondria viability) in the absence of nicotine. Nicotine control decreases cell proliferation and viability dose-dependently and is cytotoxic compared to the PG/VG controls. Nicotine (12 mg/mL) containing captain black cigar e-liquid showed more cytotoxicity (MTT assay) compared to captain black cigar alone. Nicotine-free e-liquids inhibit cell proliferation and viability in a dose-dependent manner. Nicotine-free kola, hot cinnamon candies, and menthol tobacco significantly decrease viability (calcein assay) than vehicle control.	(Rowell et al. 2017)
Human adenocarcinoma alveolar basal epithelial cells (A549 cells)	Balsamic flavor (with and without nicotine)	50 min aerosol exposure followed by analysis at 2-h, 6-h, 24-h.	Nicotine concentration not specified	No nicotine alone control present	Nicotine-free balsamic aerosol caused a significant, progressive, time-dependent loss of viability and increased cytotoxicity following LDH assay. Nicotine-contained e-liquids compared to flavor alone caused a significant loss in viability and enhanced LDH release. Quantitative and qualitative responses are superimposable to that of cigarette smoke exposure. Humectants alone did not cause cytotoxicity even 24 hours after treatment.	(Cervellati et al., 2014)
Human airway epithelial cells (H292) AND human lung fibroblast cells (HFL-1)	Tobacco Cinnamon roll Grape	30 s interval with a 4 s pulse for different time durations 5, 10, and 15 minutes	0, 6, 16, 18 and 24 mg	No nicotine alone control present	Nicotine-free tobacco and cinnamon roll-flavored e-liquid increased the secretion of inflammatory cytokines IL-6 and IL-8 in H292 and HFL-1. Nicotine-contained tobacco flavored e-liquid had a significant increase in IL-8 secretion in a dose-dependent manner in HFL-1 but not on other markers.	(Lerner et al., 2015)
Human adenocarcinoma alveolar basal epithelial cells (A549)	Tobacco Menthol	Six puffs were dissolved per 1 mL of A549 culture medium, referred to as 6 total-puff- equivalents (TPE) of aerosol, and grown in control or treatment medium for 3–8 days.	48 mg/ml (No nicotine free e-liquids present)	No nicotine control	Both EC liquids and aerosols induced epithelial-to-mesenchymal transition (EMT) Both tobacco and Menthol flavors alter cell-to-cell junctions induced an EMT, internalization of E-cadherin, increased motility, and upregulation of EMT markers. The EMT was concurrent with the plasma membrane to nuclear translocation of active β-catenin. Due to the lack of nicotine controls, it is unclear whether the effects observed are a result of an interaction between flavor and nicotine or as a flavor effect. No cytotoxicity was measured.	(Zahedi et al. 2018)
Primary human epithelial cells (HBEC), cancerous human bronchial epithelial cells CALU-3	Banana Pudding (BP)	5–25 puffs	0 and 12 mg/ml nicotine	No nicotine alone control, but 0 mg/mL BP	BP acutely elevates cytosolic Ca^2+^ in both pulmonary cell types in no nicotine samples. No adverse effects induced by PG:VG in HBEC and CALU-3. Nicotine increased ER Ca^2+^ in CALU-3. Effect of nicotine on HBEC was not characterized.	(Rowell et al., 2020)

**Table 5.** Chemical composition analyses of flavored EC.

E-liquid flavor	Experimental approach	E-liquid Nicotine Dose	Main findings	Reference
Menthol, Vanillin, Maltol	Particle size distribution	18 mg/ mL of nicotine	Vanillin and nicotine increase e-cigarette particle size distribution. Increasing the glycerol (VG) proportion in e-liquid increases EC particle size.	(Lechasseur et al., 2019)
Menthol	GC−MS Analysis of Liquid, Vapor, and Particulate Phases, FTIR Spectrometry	18 mg nicotine/mL	EC emissions contained acetaldehyde, acrolein, benzaldehyde, benzene, toluene, ethylbenzene, and xylene The concentrations of these chemicals increased with increasing VG composition. Benzaldehyde, naphthalene, diphenyl ether, menthol, and nicotine were also detected in the aerosol condensates.	(Ooi et al., 2019)
Strawberry, Cinnamon, Sweet cream	Hydroxyl radicals in reaction mixes were measured based on the formation of 2-hydroxyterephthalic acid (2OHTA)	3, 12, 24 and 36 mg/mL	VG e-liquids generated higher •OH levels than PG e-liquids, as did flavored e-liquids relative to nonflavored e-liquids (cinnamon>sweet cream>strawberry>non-flavored). E-vapor, combined with ascorbic acid, can also induce •OH formation. The levels of •OH in 3 and 12 mg/ml nicotine solutions were 19.6% and 10.0% lower than that in the equivalent nicotine-containing-vapor solutions. Conversely, 24 and 36 mg/ml nicotine solution induced 40% more •OH than the equivalent e-vapor solutions.	(Son et al., 2019)
51 flavors	Mass spectrometry	5% nicotine	At least one flavoring chemical was detected in 47 of 51 unique flavors tested. Diacetyl was detected above the laboratory limit of detection in 39 of the 51 flavors tested, ranging from below the limit of quantification to 239 μg/EC. 2,3-Pentanedione and acetoin were detected in 23 and 46 of the 51 flavors tested at concentrations up to 64 and 529 μg/EC, respectively.	(Allen et al., 2016)
Mango, Banana, Tobacco, Cappuccino, Chocolate, Bubblegum, Peppermint, Cinnamon, Cherry, Apple	GC-MS		Vanillin was the most frequently detected flavoring chemical. It was found in banana, tobacco, cappuccino, chocolate, bubblegum, and cinnamon. Chocolate and cinnamon e-liquids had the most complex flavoring profile, with 14 and 15 flavoring peaks, respectively. Tobacco flavor had the least complex flavoring profile with only 2 peaks.	(Ween et al. 2020b)

Most *in vitro* investigations on cells were carried out on at least two different cell lines belonging to one of the three classes of the cell line: primary (5 articles), immortalized normal (15 articles), and cancerous pulmonary cells (13 articles). Therefore, we have subdivided the reporting of these papers based on the cell lines used in Tables 2, 3 and 4. The mint/menthol (10 articles) was the most frequently reported to demonstrate harmful effects, followed by cinnamon flavor (9 articles) and strawberry (5 articles) in cytotoxicity assays, metabolic activity assays, and pro-inflammatory biomarkers assays.

Similarly, cinnacide (cinnamon-contained), black licorice, banana pudding, vanilla, and tobacco-induced differential toxicity *in vivo* (Table 6). Toxic effects observed *in vivo* include but are not limited to increased levels of pro-inflammatory cells in bronchial alveolar lavage (BAL), MUC5AC (the predominant mucin glycoprotein produced by respiratory epithelium that normally protects against infection but when over-expressed is associated with respiratory diseases like asthma and COPD) production, and oxidative stress markers. There were no human studies where the effects of flavors on the respiratory system were investigated.

**Table 6.** *In vivo* studies of toxicological effects of EC flavors.

Strain/sex	Exposure	E-liquid Nicotine Dose	Flavor(s) investigated.	Main findings	Reference
Adult Balb/c/ (male and female)	30 min twice daily, 6 days/week exposed for 18 days (aerosol exposure)	0 mg/mL &12 mg/mL	Black licorice (BL) Kola Banana Pudding Cinnacide	House dust mite (HDM) challenged mice exposed to nicotine-free cinnacide reduced airway inflammation and increased peripheral airway hyperresponsiveness compared to air-only controls. HDM-challenged mice exposed to flavored EC without nicotine had significant but heterogeneous effects on features of allergic airways disease compared to air controls. Nicotine-free BL increased macrophage, eosinophils, and macrophages count in bronchoalveolar lavage (BAL). Nicotine in all flavors induced an increase in macrophages and eosinophils in BAL compared to nicotine-free. Sex-difference in toxicity were not explored.	(Chapman et al. 2019)
C57BL/6 /female	2 h a day, 7 days a week, for 6 weeks	No nicotine	Vanilla	Exposures to VG/PG + vanilla increased lung tidal and minute volumes and tissue damping. Increased number of dendritic cells, CD4 + T cells, and CD19 + B cells in the lungs in the VG/PG-exposed group compared to air, irrespective of the presence of vanilla. A > 3-fold increase of 2-arachidonoylglycerol (2-AG), an anti-inflammatory mediator, and a 2-fold increase of 12-hydroxyeicosatetraenoic acid (12-HETE), an inflammatory mediator, following VG/PG exposure, with or without vanilla The PG/VG EC aerosol dysregulated genes related to biotransformation (Aldh8a1), transcription factors expressed in pulmonary surfactant (F2), synuclein-alpha (snca), which interacts with phospholipids and proteins, as well as IL-6 compared air controls.	(Szafran et al. 2020)
Balb/c/Pregnant and offspring	14–31 days exposure. Vaping topography profile of 3-s puff duration and a 55-mL puff volume every 30s.	36 mg/ml Nic (No 0 mg/mL available)	Cinnamon-flavor	Preconception and prenatal exposures to EC aerosols significantly decreased the offspring birth weight and body length compared to non-exposed controls Preconception exposure caused downregulation of 7 inflammation-related genes. Four genes were common to both dams and fetuses. Preconception EC exposure led to offspring with significantly increased lung tissue fraction at birth compared to unexposed controls. Increase in nicotinic receptors gene expression (α7nAChR) in the lungs.	(Noël et al. 2020a)
C57BL6/male	Whole-body exposure for 3 days or 4 weeks. Puff volume was 20 ml. Mice were exposed to CS or EC 4 times a day with 30-min smoke-free intervals.	18 mg/ml (No 0 mg/mL control)	Tobacco flavor	Increased BAL cellularity, MUC5AC production, and oxidative stress markers in BAL and lung compared to unexposed controls. In many cases, increases were more than for CS. BAL markers increased significantly in PG/VG + tobacco flavor + nicotine compared to PG/VG + nicotine.	(Glynos et al. 2018)
Crl:CD (Sprague-Dowley) rats/male and female	90-day nose-only inhalation to 3.2, 9.6 and 32 mg/kg/day of aerosol, which are referred to as low, mid, and high, respectively.	20 mg Nic	PG:VG control (77% PG: 23% VG) Formulation 1 (22.5% VG, 75.5% PG, 20 mg Nic) Formulation 2 (18.1% VG, 62.3% PG, 20 mg NIC and 17.6% proprietary flavor)	The inflammatory effects (increase in neutrophils, lymphocytes) in a dose-related manner were observed in the PG:VG control in both males and female groups. No flavor-dependent effect was observed for pulmonary toxicity. No nicotine-related pulmonary effects were observed.	(Werley et al. 2016)

## Discussion

This systematic review screened literature to determine which e-liquid flavors and flavoring agents (if any) elicit adverse effects in the airways. The studies identified several flavor categories demonstrating toxicity (Tables 2, 3 and 4), suggesting that several flavor chemicals might adversely affect respiratory cells. However, the most frequently reported e-liquids eliciting adverse responses were cinnamon, menthol, and strawberry flavors. It is unclear whether these flavors are the most frequently reported toxic as these are the most investigated. Further, it is unclear which flavoring chemical plays the critical role in the toxicity of strawberry-induced pulmonary cells; nonetheless, cinnamaldehyde and menthol are the primary components of cinnamon and menthol flavors, respectively (Clapp et al. 2017; Omaiye et al. 2019), which were found to activate TRPS. Several investigators showed induction of oxidative stress, inflammation and disruption of pulmonary barrier functionalities following e- liquids flavor exposure (Behar et al. 2014; Clapp et al. 2019, 2017; Lamb, Muthumalage, and Rahman 2020; Muthumalage et al. 2019, 2018). The mode of toxicological actions for many of the flavors in e-liquids is elusive. Still, evidence suggests that flavors such as cinnamon, menthol, strawberry, tobacco, and many others either induce one or more of the following adverse effects: mitochondrial dysfunction, cell death, ROS production, and dysregulation of inflammatory cytokines (Tables 2, 3 , 4 and 6). Given that cinnamaldehyde and menthol might activate TRPA1 and TRPM8, respectively, the adverse effects observed attributed to cinnamon and menthol may occur via the TRPs. Nonetheless, a few omissions were identified such as (1) absence of appropriate controls, (2) lack of validation for the experimental model, and (3) justification for concentrations used, which may impact inter-study comparisons and variability and hinder accurate interpretation of results. As such, it is recommended that experimental approaches be employed to reduce discrepancies in the toxicity testing of EC flavorings in future studies.

### Adverse effects of flavored e-liquids

**Figure 3.**
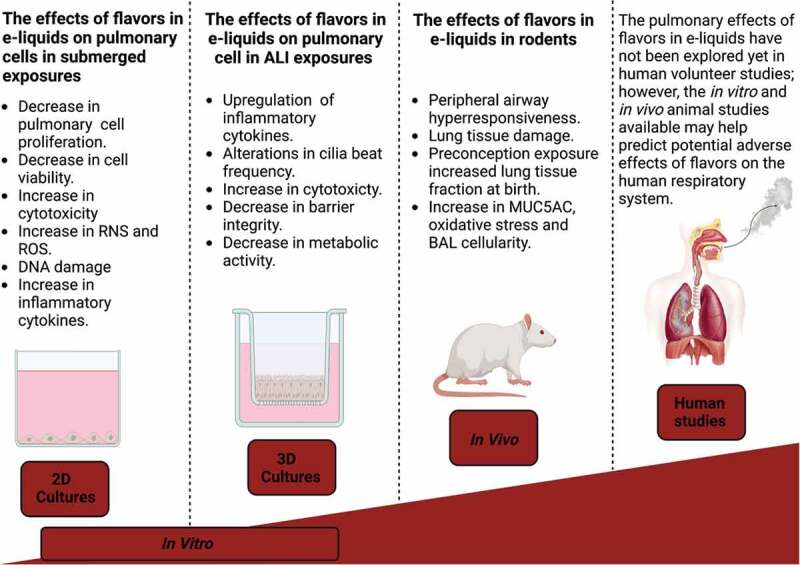
The effects of flavors in e-liquids reported in different experimental models. The figure was produced with Biorender.com.

The evidence presented in this review suggests that flavors in e-liquids are not non-hazardous. Evidence regarding the risk of e-liquids might be biased or affected due to the limited number of appropriate toxicological studies. The most frequently reported adverse effects are reduced cell count and viability, altered pro-inflammatory biomarkers, cytokine release, and increased oxidative stress (Figure 3 for a schematic overview). These effects mentioned are consistent across pulmonary cell types, including normal and cancerous cells, large and small airway epithelial cells, alveolar epithelial cells, pulmonary smooth muscle, fibroblasts, and alveolar macrophages. Although there are no apparent studies where the sensitivity to different flavored e-liquids across primary, normal immortalized, and cancerous cells are simultaneously probed; nonetheless, in this review, primary cells and normal immortalized cells are seemingly more susceptible to the adverse effects of flavored e-liquid exposures than cancerous cell lines (Tables 3 and 4) (Leslie et al. 2017; Pinkston et al. 2020).

Immortalized human cell lines are normal cells artificially manipulated to proliferate indefinitely. In contrast, cancer cell lines are transformed cells with mutations in oncogenes and tumor suppressor genes. There is evidence to suggest that cancer cells have increased levels of antioxidant enzymes such as superoxide dismutase and catalases compared to healthy cells, potentially as a defense mechanism against oxidative stress or to promote DNA damage and cancer progression (Asaduzzaman Khan et al. 2010; Chung-man Ho et al. 2001; Sun et al. 1989). Given the differential expression of antioxidant enzymes in cancer cells vs. primary and normal cells, this may contribute to the inter-studies variability conducted on EC flavors toxicity. Therefore, caution needs to be taken in interpreting and drawing conclusions regarding findings obtained from experimental models that may not reflect normal human cellular physiology.

Currently, there is no standardized protocol for physiologically relevant EC exposure dosage ranges *in vitro* or *in vivo*. Most studies carried out dose-response investigations (Behar et al. 2018a; Bengalli et al. 2017; Ganguly et al. 2020; Leslie et al. 2017; Lucas et al. 2020; Rowell et al. 2017). Decrease in cell proliferation and viability are indicative of increased cytotoxicity. A concentration-dependent decrease in cell proliferation and cell viability was observed (Tables 2, 3 and 4) with a mixture of tobacco, coconut, vanilla, and cookie (Lucas et al. 2020), hot cinnamon, menthol tobacco, black cigar (Rowell et al. 2017), butter finger and caramel (Behar et al. 2018a) following cellular exposure. Further, a combination of single-dose and multiple-dose studies reported that menthol (Czekala et al. 2019; Lamb, Muthumalage, and Rahman 2020; Muthumalage et al. 2019), blueberry and tobacco (Czekala et al. 2019), cinnamon (Behar et al. 2014; Rowell et al. 2017), menthol tobacco and kola (Rowell et al. 2017), and creamy/buttery and caramel (Behar, Wang, and Talbot 2018b) demonstrated the most significant effects were mitochondrial dysfunction in airway epithelial cells suggesting some role for induction of mitochondrial-induced apoptosis. In addition, cinnamon (Noël et al. 2020b), menthol (Muthumalage et al. 2019), and crème brûlée flavor (Pinkston et al. 2020) showed the most significant increase in production of markers of oxidative stress such as reactive oxygen species (ROS) and reactive nitrogen species (RNS) in bronchial epithelial cells (Figure 2). ROS and RNS are highly reactive chemical molecules intrinsic to cellular functioning when maintained at low normal cell levels. Still, excess ROS and RNS formation induces damage to cellular macromolecules, including DNA affecting normal cell functions (O’Farrell et al. 2021).

Several studies investigated the ability of flavored e-liquids to induce differential expression of pro-inflammatory cytokines in pulmonary epithelial cells (Clapp et al. 2019; Pinkston et al. 2020; Ween et al. 2020b), basal cells (Zahedi et al. 2018), and lung fibroblasts (Behar et al. 2014; Lucas et al. 2020; Omaiye et al. 2019b). Although Bahl et al. (2012); Leigh et al. (2016); Lucas et al. (2020); Rowell et al. (2017) explored different ranges of flavored e-liquid concentration, none of these justified the relevance of the applied dosages concerning human exposure. Lucas et al. (2020) investigated the concentration-dependent effects on IL-8 release but found that the highest concentrations of flavor e-liquid (0.5% or 1%) did not induce a marked change in IL-8 whereas 0.25% elevated the levels. It is unclear why this ‘bell-shaped’ effect was noted with a mixture of tobacco, coconut, vanilla, and cookie. However, it might be that higher concentrations failed to induce a rise in IL-8 as a consequence of enhanced cytotoxicity (Lucas et al. 2020). IL-8 is a potent chemokine that is critical in leukocyte infiltration during inflammation. IL-8 levels were elevated by chocolate, banana, cinnamon, tobacco, and a mixture of tobacco, coconut, vanilla, and cookie flavors in lung epithelial cells and pulmonary fibroblast. In Tables 2, 3 and 4, menthol, cinnamon, cool cucumber, classic menthol, and caffe’ latte flavored e-liquids produced increased release of pro-inflammatory biomarkers (IL-1ß, IFN-γ, IL-17) in lung epithelial cells. Coffee enhanced the release of IL-6, CXCL1, and CXCL2, whereas strawberry elevated the release of IL-1ß, IL-10, CYCL1, CXCL2, and CXCL10. IL-1ß, CXCL1, and CXCL2 which are pro-inflammatory cytokines (Leigh et al. 2016). IL-10 is an anti-inflammatory cytokine, and IL-6 functions as either pro- or anti-inflammatory depending upon the phase of inflammation at which it is released. In contrast, in a separate study, apple flavor reduced the pro-inflammatory cytokines secretion of TNF-α, IL-6, IP-10, MIP-1α, and MIP-1β (Ween et al. 2020b). Tables 2, 3 and 4 show that EC liquid flavors increased pro-inflammatory biomarkers, predominantly in pulmonary epithelial and fibroblast cells. The release of cytokines by the lung epithelium leads to recruitment, activation, and differentiation of innate and adaptive inflammatory cells: macrophages, neutrophils, B cells, and T cells. Although not many investigators assessed flavor-induced pro-inflammatory cytokines in immune cells *in vitro*, cinnamon suppressed alveolar macrophage phagocytosis *in vitro* (Clapp et al. 2017), suggesting prolonged exposure to cinnamon flavor may impact the resolution of lung inflammation. The proposed mechanism for this effect is elusive but likely via might involve one its predominant constituents, cinnamaldehyde. The latter was found to inhibit the toll-like receptor (TLR) subtype 4 activation (Youn et al. 2008), which subsequently might play a key role in macrophage phagocytosis (Skjesol et al. 2019). In alveolar macrophages, phagocytosis is critical for (1) defense against invading pathogens, (2) removal of dead cells or foreign particles, and (3) resolution of inflammatory responses and tissue remodeling, processes that are mediated by various surface receptors, including TLRs (Micera et al. 2016; Paradowska-Gorycka 2015). Evidence suggests that the resolution of respiratory inflammation may be altered in users of cinnamon flavors.

*In vitro* observations following flavored-e-liquid exposures were mirrored in the few *in vivo* animal studies on EC-mediated toxicity. Cinnacide, whose active flavor ingredient is cinnamaldehyde, and black licorice produced alterations in airways inflammation *in vivo* (Chapman et al. 2019) (Table 6) by lowering numbers of eosinophils and increasing number of macrophages, respectively, in BAL in allergenic airways disease utilizing adult Balb/c mouse model (males and females). In contrast, vanilla elevated number of dendritic cells, CD4+ cytotoxic T cells, and CD19 + B cells infiltration in the C57BL/6 mouse (female) lungs (Szafran et al. 2020). Further, the effects of cinnamon-flavor exposure in Balb/c pregnant mice and their offspring showed that preconception flavor exposure increases lung tissue fraction at birth compared to non-exposed groups (Noël et al. 2020a), suggesting that cinnamaldehyde may be teratogenic. Glynos et al. (2018) noted a flavor effect on broncoalveolar lavage fluid (BALF) markers, such as elevation in malondialdehyde and BALF protein carbonyls levels, increased BALF macrophages number, elevated IL-1β and IL-6 levels, and rise in BALF total cell count in an acute C57BL/6 male mice exposure. Werley et al. (2016) demonstrated that addition of flavor (flavor type unknown) did not impact pulmonary toxicity in a chronic Sprague-Dawley rat (female and male) exposure study. However, the effects observed were driven by the PG/VG vehicle (Table 6). Taken together, the reported *in vivo* animal studies suggest potential adverse lung effects for at least some flavored-e-liquids on the pulmonary system in rodents irrespective of strain and/or gender.

It is recognized that caution needs to be taken in interpreting the *in vivo* studies on EC-initiated toxicity (Marczylo 2020) where whole-body exposures were performed. Animal experimentation does not mimic human exposure models as these rodents are exposed through inhalation and the oral route via grooming. Further *in vivo* studies examining the influence of different flavors are warranted to enable for comparisons between flavors. The above evidence points toward potential harm from exposure to EC flavors. Some e-liquids induce oxidative stress, release of pro-inflammatory mediators, and cytotoxicity (Figure 3), which may exert implications for healthy lungs and might exacerbate inflammatory-mediated lung diseases such as COPD (O’Farrell et al. 2021), emphysema, and pneumonia. However, some uncertainties need addressing. Some studies did not provide sufficient detail on exposure dose, nicotine concentrations, and exposure durations to enable investigations to be reproduced. In addition, most studies (Table 2, 3 , 4 , 5 and 6) were not designed to include appropriate controls (PG/VG, nicotine) to enable an assessment of the specific contributions of flavor components to observed toxicity. These findings are important because studies, where PG/VG and nicotine controls were included, showed that these alone exert some adverse consequences that may account for at least some of the observed toxicities of the flavored e-liquids.

### Additive effects of nicotine and PG/VG on flavor-induced toxicity

Nicotine is a potent stimulator of cell proliferation and may stimulate cancer development and growth (Dasgupta 2006; Khalil et al. 2013; Lee et al. 2005; Mravec et al. 2020). Nicotine is an agonist for the nicotinic acetylcholine receptors (nAChRs) (Dani 2015; Victoria et al. 2022), which are functionally expressed in the non-neuronal tissues of the lung (Chernyavsky et al. 2015; Improgo, Tapper, and Gardner 2011;). There are more than a dozen different nAChR subunit proteins, subdivided into α and β subfamilies, which form pentameric ion channels consisting of either a single type of α subunit (homopentamers) or a combination of α and β subunits (heteropentamers) (Shahsavar et al. 2016). As ligand-gated ion channels, nAChRs undergo complex allosteric changes in response to binding either the endogenous ligand acetylcholine (Ach) or exogenous ligands, including nicotine. nAChRs are classically linked to the plasma membrane depolarization required for neurotransmission; however, non-neuronal nAChRs in the lung act most frequently as calcium channels and were reported to activate numerous cellular pathways upon binding to either adrenergic receptors, nicotinic receptors, or by direct action within the cytoplasm (Wen et al. 2011) which regulate cell proliferation. Nicotine alone is not a carcinogen but is a tumor promoter (Ping Wu 2019). High doses of nicotine induce multi-organ toxicity and perhaps death from paralysis of respiratory muscles via the nAchRs (Mishra et al. 2015).

Most commercially available e-liquids contain nicotine; therefore, it is understandable that most studies report using nicotine-containing products (Voos, Goniewicz, and Eissenberg 2019). There is conflicting evidence regarding the additive effects of nicotine to flavor-induced toxicity. Only 4 (Bahl et al. 2012; Leigh et al. 2016; Rowell et al. 2017; Ween et al. 2020a) out of the 28 *in vitro* studies in Tables 2, 3 and 4 that reported adverse flavor effects in nicotine-containing e-liquids had included appropriate controls for both nicotine content and PG/VG to isolate flavor-effects. (Leigh et al. 2016) and (Bahl et al. 2012) showed that nicotine exerted no additive effects on strawberry, coffee (Leigh et al. 2016), menthol, and cinnamon (Bahl et al. 2012) flavor-dependent toxicity in H929 cells and HPF cells, respectively. In contrast, (Rowell et al. 2017; Ween et al. 2020a) noted the effects of nicotine-alone controls on cell proliferation inhibition, decreased cell viability flavor (Rowell et al. 2017a), and necrosis by Sytox Green (Ween et al. 2020a) in Calu3 and NHBE cells, respectively. These discrepancies may arise from 1) variability in nicotine concentrations and/or 2) differential expression of nAchRs or β-adrenergic receptors in the submerged models used. Because nAChR activation often leads to a positive feedback loop that induces receptor expression, high-concentration stimulation of nAChRs might lead to channel desensitization and decreased activity. Thus, elucidating functional roles for nAChRs is particularly complex and requires consideration of subunit composition, dose response, and duration of ligand stimulation. Further, undifferentiated bronchial epithelial cells in submerged cultures may be considered uniformly basal. In contrast, validated ALI models of bronchial epithelium represent a model of differentiated airway cells, including ciliated epithelial cells and goblet cells. Therefore, the lack of a nicotine effect in submerged cell cultures may be attributed to low expression levels or absence of cell surface receptors, including nAChRs and β-adrenergic receptors. Ganguly et al. (2020) reported additive effects of nicotine in reducing inflammatory levels of cytokines of IL-1ß, 1 L-10, IL-13, and IL-6 in a validated NCI-H441 ALI model, which probably expressed nAChRs. In contrast, (Wang et al. 2020) showed that *in vivo* EC exposure with or without nicotine affected lung inflammation, repair responses, and extracellular matrix remodeling mediated by the α7 nicotinic acetylcholine receptor (nAChRα7) in a gender-dependent manner. In addition, Glynos et al. (2018) detected no nicotine-dependent effects on inflammatory markers (IL-6, IL-1β, TNF-α) release and MUC5AC score in acute or chronic C57BL/6 mouse exposure models. However, Glynos et al. (2018) noted a nicotine plus flavor-dependent effect suggesting that e-liquid toxicity may not be associated with nicotine but rather the flavor content. No nicotine-related adverse pulmonary effects were observed in male or female Sprague-Dawley rats following chronic exposures, but a PG/VG effect was observed. Although there is contrasting evidence on the adverse health effects of nicotine in *vitro* against *in vivo* EC studies, the non-harmful effects of nicotine via inhalation were proven in a two-year survey by Waldum et al. (1996). Therefore, evidence indicates that nicotine may promote EC-related adverse pulmonary effects when these are initiated by the flavor or vehicle component of the e-liquid.

Four studies enabled assessment of PG/VG components alone by including that as vehicle control. Evidence showed that PG/VG-alone decreased metabolic activity in H929 (Leigh et al. 2016), H441, and HBECs (Woodall et al. 2020) via a mechanism that likely involves the blockade of glucose transporter uptake. Woodall et al. (2020) showed that PG/VG reduced glucose uptake and metabolism via the glucose transporters (GLUT1, GLUT2, and GLUT10) in human bronchial epithelial cells. In comparison, Bahl et al. (2012), Leigh et al. (2016), and Ween et al. (2020a) reported that PG/VG exerted no marked adverse effects on the pulmonary cells. Therefore, based on the contrasting evidence provided above, potentially stemming from methodological differences including the choice of cell line and dosage, attributing the adverse effects observed with flavored nicotine-contained e-liquids to the presence of nicotine by comparison to their nicotine-free e-liquid equivalents (Table 2, 3 , 4 , 5 and 6), without appropriate nicotine- and PG/VG-alone control may be misleading.

As such, future studies on flavors and/or flavoring ingredients of e-liquids might be improved by including appropriate controls: (1) cell-only for submerged exposures and air-only controls for air-liquid interface (ALI) exposures, (2) PG/VG/vehicle alone controls, and (3) PG/VG + nicotine controls to better inform on the contributions of the flavor compounds. In addition, justifications for e-liquid dosages might improve the interpretation of the relevance of the findings for health of individuals that vaper. These would lead to a better understanding of the relative contributions of flavor compounds, vehicles, and nicotine to the observed adverse effects of e-liquids and aid the ongoing debate over the safety and banning of flavored-e-liquids.

### Recommended approach to reduce uncertainty in EC investigation in vitro and in vivo models

This review note the following: 1) lack of appropriate controls in many studies to enable reliable interpretation of data for identification of hazards and risks due to flavor components, 2) ambiguity over physiologically relevant doses of e-liquid/flavor used, and 3) lack of assessments of multiple dosing to establish the presence of dose-dependent relationships. Therefore, this section proposes an approach to reduce discrepancies among EC investigations, whether *in vitro* or *in vivo*.

*In vitro* submerged culture models are extensively employed in EC-induced toxicity screening. Evidence suggests that cytotoxicity of e-liquids in submerged models accurately predicts aerosol cytotoxicity 74% of the time (Behar, Wang, and Talbot 2018b; Sassano et al. 2018). Behar, Wang, and Talbot (2018b) conducted a pattern of cytotoxicity study on 35 e-liquids. The patterns were characterized as follows: (1) both the refill fluid and its aerosol were non-cytotoxic (7 of 35 = 20%) (2) both the refill fluid and its aerosol were cytotoxic (19 of 35 = 54%) (3) the refill fluid was cytotoxic, but the aerosol was not (1 of 35 = 3%) (4) the aerosol was cytotoxic, but the refill fluid was not (8 of 35 = 23%) Collectively when combining patterns (1) and (2), 74% of the 35 unheated refill fluids correctly predicted cytotoxicity of their corresponding aerosol. The remaining 26% did not reliably predict cytotoxicity because: (1) certain flavors (i.e., menthol artic) were cytotoxic in submerged cultures but not in their aerosol exposures in the ALI model (2) other flavors are cytotoxic in their aerosolized form (i.e., butter finger and caramel) but not in their liquid form (Behar, Wang, and Talbot 2018b). Although primary human bronchial epithelial cells in submerged cultures are basal cells and do not recapitulate the microenvironment of *in vivo* lung epithelial structures, using e-liquid with submerged models with the inclusion of appropriate controls: nicotine-alone, PG/VG-alone, and physiologically relevant range of flavor doses may help high-throughput screening of the extensive library of flavored e-liquids for toxicity. Subsequently, the most revealing e-liquids might be progressed into aerosol exposures using air-liquid interface (ALI) systems with standardized puff profiles suggested by CORESTA (CORESTA 2015) to eliminate potential variability within EC vapor toxicity.

In contrast to the submerged models, ALI lung cell models that have been validated for adequate transepithelial membrane resistance (TEER) measurements, expression of differential markers for goblet cells (MUC5AC), ciliated cells (FOXJ1), Clara cells (SCGB1A1), and basal cells (KrT5) may be used to investigate physiological and pathophysiological responses of the respiratory tract, molecular events, modes of action and interactions of different cell types. Potentially, these cell models mimic the *in vivo* microenvironment of cells in the respiratory tract more closely, namely, being apically exposed to air. In addition, these cells generate airway surface liquid (ASL), and the apical surface may be exposed directly to EC vapor. It is probably a better model than adding e-liquid in submerged basal cell culture. Such ALI models focus on anatomical regions of the lung or molecular pathways and aim to model relevant processes *in vivo*. Pairing this with analytical approaches to identify potential chemical compositions and concentrations (especially in flavors where toxicity is observed) for further investigation may eventually lead to limits on specific flavor chemical concentrations or outright bans from e-liquids. In addition, studies assessing the usefulness of ECs in risk and harm reduction needs to consider comparing e-cigarette data against conventional cigarettes before concluding the relative safety of these devices. This could be a gold-standard protocol to screen e-liquids toxicity that minimizes uncertainties.

To date, no apparent human studies have explored the toxicological effects of flavors in e-liquids on the respiratory system. Nonetheless, results such as inflammatory biomarkers, gene expression and cytotoxicity from either *in vitro* studies where the experimental model used to assess the safety of EC flavors closely recapitulates the microenvironments of the 3D human lung (ALI, or lung-on-chip) or *in vivo* animal testing may help discuss the potential extrapolation of EC toxicity to humans, taking into consideration dosage and exposure times required to mimic human exposures (Figure 3).

## Conclusions

Some flavors in e-liquids may elicit pulmonary toxicity. However, these flavors are generally believed to attract long-time tobacco cigarette smokers to switch to EC use. As such, imposing restrictions on the use of flavors based upon reported evidence may be counterproductive. This is because 1) several *in vitro* studies demonstrated that EC-mediated toxicity to be related to PG/VG and nicotine rather than flavors per se, 2) not all flavors produce toxicity, and 3) more evidence-based risk assessments need to be used for those flavors that induced some toxicity to set concentration limits to reduce the potential for causing harm in humans. Although no apparent human studies data accounted for potential adverse pulmonary effects of flavored e-liquids, *in vitro* and *in vivo* studies reported toxicity attributed to cinnamon, strawberry, menthol, and other flavors. Therefore, public health authorities need to extrapolate data from these studies to predict risks posed by flavors to human health.

## Data Availability

The authors confirm that the data supporting the findings of this study are available within the article.
